# Three-dimensional nanophotonics with spatially modulated optical properties

**DOI:** 10.1038/s41377-025-02166-5

**Published:** 2026-03-03

**Authors:** Yannick Salamin, Gaojie Yang, Brian Mills, André Grossi Fonseca, Charles Roques-Carmes, Quansan Yang, Justin Beroz, Steven E. Kooi, Marc de Miguel Comella, Kiran Mak, Sachin Vaidya, Daniel Oran, Corban Swain, Yi Sun, Shai Maayani, Jamison Sloan, Amel Amin Elfadil Elawad, Josue J. Lopez, Edward S. Boyden, Marin Soljačić

**Affiliations:** 1 Research Laboratory of Electronics, MIT, Cambridge, MA USA; 2 Department of Physics, MIT, Cambridge, MA USA; 3 CREOL, The College of Optics and Photonics, University of Central Florida, Orlando, FL USA; 4 McGovern Institute for Brain Research, MIT, Cambridge, MA USA; 5 Ginzton Laboratories, Stanford University, Stanford, CA USA; 6 Department of Mechanical Engineering, MIT, Cambridge, MA USA; 7 Institute for Soldier Nanotechnologies, MIT, Cambridge, MA USA; 8 CFIS, Universitat Politècnica de Catalunya, Barcelona, Spain; 9 Irradiant Technologies, Cambridge, MA USA; 10 Department of Biological Engineering, MIT, Cambridge, MA USA; 11 Department of Brain and Cognitive Sciences, MIT, Cambridge, MA USA; 12 Howard Hughes Medical Institute, MIT, Cambridge, MA USA; 13 K. Lisa Yang Center for Bionics, MIT, Cambridge, MA USA; 14 Center for Neurobiological Engineering, MIT, Cambridge, MA USA

**Keywords:** Photonic crystals, Laser material processing, Nanophotonics and plasmonics

## Abstract

Nanophotonics has revolutionized the control of light-matter interactions in various fields of fundamental science and technology. In this work, we propose Implosion Fabrication (ImpFab) as a versatile nanophotonics fabrication platform providing the highest spatial resolution, material versatility, and full volumetric control. ImpFab uniquely combines top-down lithography with bottom-up nanoparticle assembly within a hydrogel scaffold, enabling precise control over optical material properties, such as refractive index, by adjusting printing parameters. We showcase the potential of ImpFab by fabricating three-dimensional photonic crystals and quasicrystals, as well as demonstrating optical structures with spatially modulated unit cell material properties. Our results highlight the potential of ImpFab in producing nanostructures with tailored optical functionalities, which are crucial for applications in sensing, imaging, and information processing, and opening new avenues in developing non-Hermitian photonic systems with spatially controlled gain and loss.

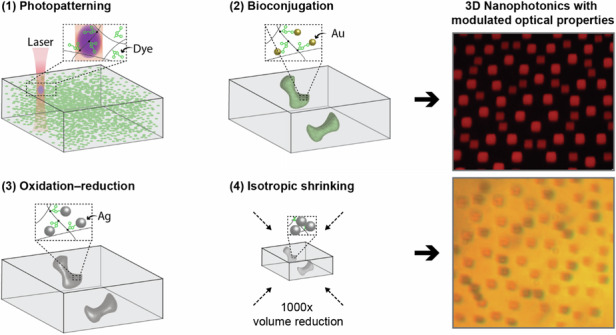

## Abstract (web summary)

We combine hydrogel photopatterning with nanoparticle growth to form silver-enhanced features that isotropically shrink 1000 × in volume to realize nanoscale 3D structures with tunable optical properties.

## Introduction

Nanophotonics has emerged as a powerful paradigm to manipulate and control light-matter interactions at the nanoscale^1–3^, opening new avenues for fundamental research and technological advancements in various fields, including sensing, imaging, and information processing^4^. Three-dimensional (3D) optical structures, such as photonic crystals, offer unique opportunities for enriching nanophotonics, as they can provide enhanced light-matter coupling^5^, as well as tunable photonic bands to explore topological physics, notably Weyl and Dirac points^6–9^. Likewise, multilayer moiré crystals have recently been shown for enhanced light localization^10^, optical singularities^11^, as well as interfacial nonlinear responses^12^. Furthermore, photonic quasicrystals have been garnering considerable attention, primarily due to their distinct physical properties and promising potential in realizing new photonic devices^13,14^.

Among exciting frontiers in nanoscale light-matter interactions, non-Hermitian nanophotonics has emerged as a rapidly growing research area, driven by the intriguing possibilities of manipulating gain and loss in optical systems to achieve novel functionalities and performance enhancements^15,16^. Such optical systems require advanced fabrication methods, capable of integrating materials with controlled properties with high spatial precision^17^. Yet, the vast majority of implementations have focused on coupled resonator systems, such as waveguide arrays^18–20^ or ring resonators^21,22^, due to fabrication challenges in creating 3D structures with spatially varying material properties.

Among the most popular approaches in 3D nanofabrication, two-photon polymerization (TPP), which relies on the photoactivation of a resin by a high-power laser^23,24^, has been a prominent method due to its versatility. Yet, TPP suffers from several major drawbacks for photonic applications, such as low-refractive-index contrast, single-material structures, limited resolution, and geometrical constraints, which impede the creation of arbitrary 3D shapes at optical wavelength scales^25^. Alternatively, bottom-up approaches have demonstrated the potential for realizing 3D crystals with various lattice geometries and materials^26,27^. Recently, an innovative approach has emerged – Implosion Fabrication (ImpFab) – employing hydrogels as 3D scaffolds for the volumetric deposition of various materials, enabling the creation of complex 3D nanostructures with nanoscale precision^28,29^.

Here, we explore the potential of ImpFab’s ability to directly assemble silver particles inside hydrogels for complex 3D nanophotonic structures with modulated optical properties. We demonstrate the nanofabrication and analyze diffraction patterns of 2D and 3D photonic crystals with diverse lattice structures. We also demonstrate aperiodic structures such as moiré crystals and quasicrystals, which lack periodicity but can have rotational symmetries forbidden in conventional crystals. A distinctive aspect of our method is its ability to manipulate the effective optical properties of the deposited material continuously in three dimensions. This paves the way for new directions in nanophotonics research, in particular, the development of sophisticated non-Hermitian nanophotonic systems with spatially controlled optical functionalities such as gain and loss.

## Results

### Implosion fabrication: 3D nanoprinting

**Fig. 1:**
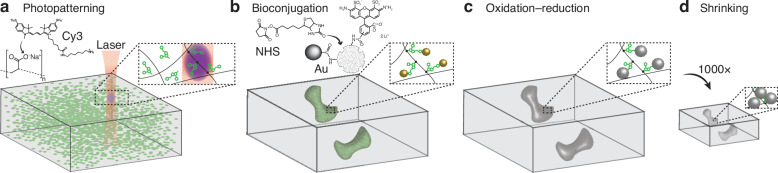
Implosion fabrication (ImpFab) method. **a** Casting of hydrogel precursor into the desired composition. Infiltrated dye molecules are covalently bound to the polymer matrix by two-photon excitation (purple voxel). Cy3: Sulfo-Cyanine3 Amine dyes. **b** Nanoparticle conjugated with the functional group of the patterned fluorescent on the backbone. NSH: Biotin-NHS; Au: Streptavidin Fluoronanogold. **c** The pattern is intensified by an oxidation-reduction process to grow larger silver particles at the gold nucleation sites. **d** Isotropic shrinkage of the hydrogel matrix and patterned 3D structure

The nanofabrication process we present in this paper, ImpFab, is depicted in Fig. 1. The approach combines top-down lithography for full control of the geometry, and bottom-up nanoparticle conjugation for the deposition and growth of various materials^28,29^. A hydrogel scaffold serves as both the 3D matrix for the incorporation of functional materials, and as a scalable structure, capable of expansion and shrinkage, facilitating the achievement of truly nanoscale (~ 50 nm) features^28^.

The process begins with the preparation of the hydrogel composed of a solution of sodium acrylate, acrylamide, and bisacrylamide^28^. Weight concentrations of each component are optimized for a volume shrink ratio of 1000 x (10 x per side). The gel is then washed in water (dH_2_O) to reach full expansion. Subsequently, the gel is immersed in a solution containing fluorescent Sulfo-Cyanine3 Amine (Cy3) dyes (250 *μ*M) for 1 h, ensuring full diffusion of dyes throughout the porous hydrogel, see Fig. 1a. Next, a laser scanning microscope is employed to selectively photo-activate a chemical binding between the fluorescent dyes and the hydrogel backbone. Infiltrated dye molecules within the laser beam voxel (highest intensity volume) are covalently bound to the polymer matrix by two-photon excitation: see inset of Fig. 1a. Additional information about the photopatterning system can be found in the Supplementary Information Section S3. Upon completion of the patterning process, the gel is thoroughly washed in water to remove the unexposed dyes, leaving behind the optically patterned 3D structure composed of the fluorescent molecules with a functional group. We then proceed with a series of molecule-nanoparticle nanoconjugation steps to introduce desired optical functionality.

In the following, we describe the process for silver nanoparticles^28^. This procedure can be generalized to a much greater library of materials of interest, including quantum materials, oxides, diamond, upconversion materials, and semiconductors^29^. The gel is first washed in a solution of 100 *μ*M N-hydroxysuccinimido biotin (NHS-Biotin) molecules, and subsequently immersed for > 8 h in a solution of streptavidin-fluoronanogold particles, see Fig. 1b. After each chemical process, the gel is thoroughly washed in deionized water (dH_2_O) to minimize unintended particles in the gel background. After a wash in 50 mM ethylenediaminetetraacetic acid disodium salt dihydrate (EDTA) for 30 min, we follow with silver intensification through an oxidation-reduction reaction at the gold nucleation sites, see Fig. 1c. In addition to dH_2_O, the gel is washed in 50 mM sodium citrate for 1 h. Finally, the gel is subjected to a controlled shrinking process, during which the embedded structures undergo an isotropic volumetric reduction, leading to the formation of the final 3D heterostructure with nanoscale features, see Fig. 1d. For the shrinking process, the gel is washed in a two-step salt solution of 0.1 M magnesium dichloride (MgCl_2_) and 0.1 M calcium chloride (CaCl_2_), each for about 20 min.

**Fig. 2:**
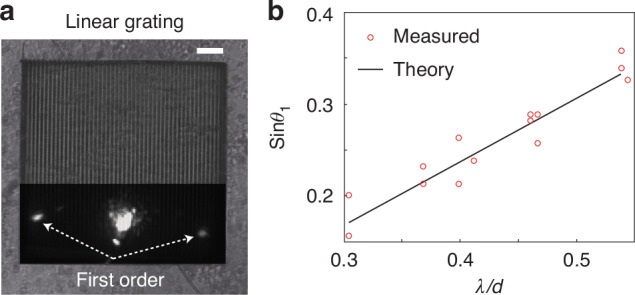
Optical response of silver diffraction gratings. **a** Optical microscopy image of a silver grating with a pitch of 850 nm. The overlapping inset shows a representative first-order optical diffraction pattern obtained under narrow-band illumination at 520 nm. Scale bar is 10 *μ*m (**b**). Measured diffraction angle as a function of the normalized ratio between wavelength (*λ*) and grating pitch (*d*). The black line indicates prediction based on the grating equation, showing good agreement with experimental data

To experimentally validate the structural controllability and optical performance, we fabricated silver diffraction gratings with groove periods ranging from 520 nm to 1700 nm. Figure 2a shows an optical microscopy image of a representative grating with a period of 850 nm. Optical characterization using a custom-built Fourier microscopy system revealed distinct first-order diffraction under narrow-band illumination, as shown in inset of Fig. 2a. The measured diffraction angles closely match the expected theoretical predictions, confirming both the accuracy of the patterned periodicity and the isotropic shrinking process. Further details on the structural integrity and process optimization can be found in the Supplementary Information Sections S1 and S2.

The method described above offers unprecedented design freedom and material versatility^28^, enabling the creation of complex 3D photonic devices for a wide range of applications. In particular, incorporating fluorescent dyes and metallic particles in combined structures enables the introduction of gain and lossy elements, an essential aspect of non-Hermitian photonics.

### Modulated optical properties

**Fig. 3:**
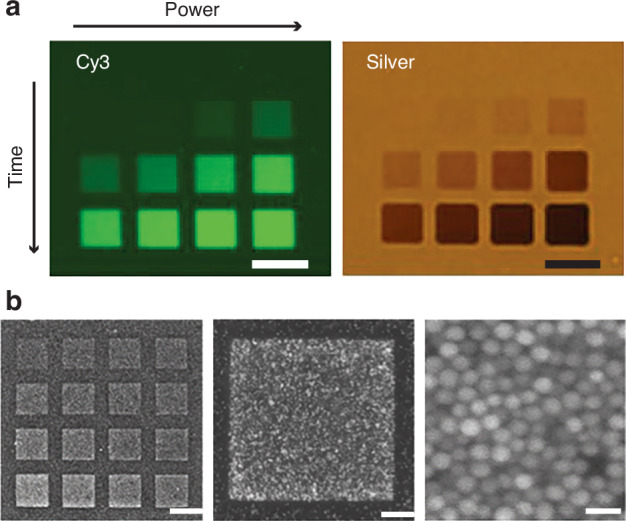
Printed molecule density gradient. **a** Square patches patterned with different laser power and dwell time. Fluorescent image of square patches post-print (left panel). The bright-field image shows the same patches after silver intensification (right panel). Scale bars are 10 *μ*m. **b** SEM images showing patterned regions with dense, uniform gold nanoparticles. (Right) High-magnification SEM image reveals interconnected nanoparticles within the patterned area, confirming high chromophore activation and efficient material deposition. Scale bar: left = 10 *μ*m, middle = 1 *μ*m, right = 50 nm

We first demonstrate how the optical properties of the printed structures can be modulated through variations in printing parameters. We printed embedded square patches with an approximate thickness of 20 *μ*m under different settings of laser power and scan speed. The left panel of Fig. 3a shows a fluorescence image of the hydrogels post-printing, where a clear gradient in the patterned molecule density can be identified. The activation of chromophores is maximized with increased laser energy, which is directly proportional to the laser power and printing dwell time. This correlation is translated to the silver density of the final structure, as observed by the pronounced contrast in the right panel of Fig. 3a, showing a wide-field microscope transmission image of the gel. This underscores our control over the optical properties of silver through adjustment of the printing parameters. Scanning electron microscopy (SEM), shown in Fig. 3b, revealed densely packed and interconnected gold nanoparticles, indicating efficient chromophore activation and uniform material deposition within the patterned regions.

**Fig. 4:**
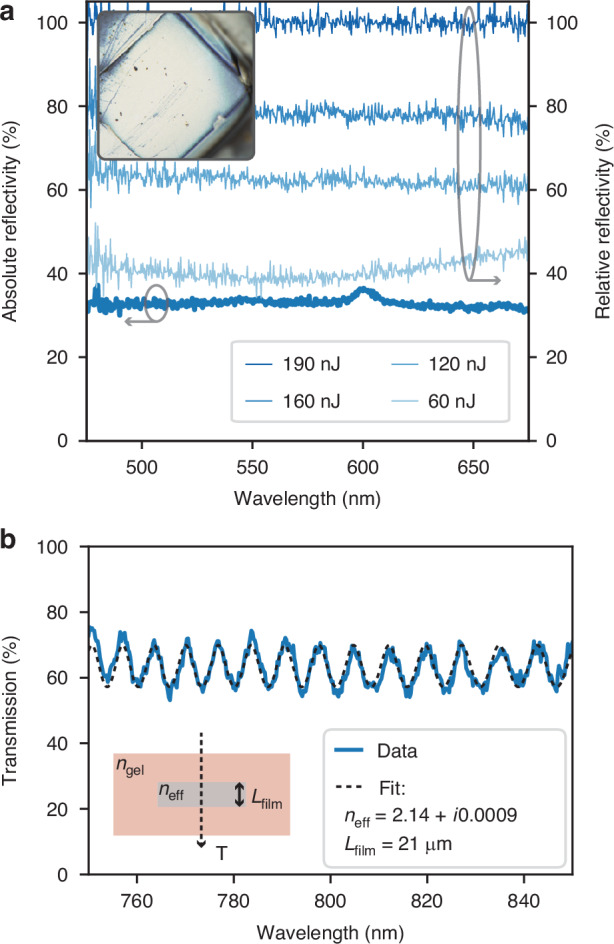
Modulation of printed material’s optical properties. **a** Absolute reflectivity of a high-density silver patch printed at the gel-air interface (left axis). The inset shows a stitched region of adjacent square patterns used for characterization. Relative reflectivity spectra for patches printed with varying laser energies (right axis), demonstrating relative reflectivity tunable over more than 50%. **b** Transmission spectrum of a low-density silver patch exhibiting Fabry-Pérot interference

A quantitative characterization of the final silver composition is obtained by reflection and transmission spectroscopy. To facilitate the characterization of the material properties, we printed a set of silver patches at the gel surface, exposing the silver-air interface. To ensure adequate area coverage, we printed four square patterns adjacent to one another (a visible stitch can be observed in the inset of Fig. 4a). The reflectivity was measured using a custom Fourier space microscope system. Figure 4a shows the measured absolute reflectivity of the printed silver under normal incidence across the visible spectrum, at its highest density. A mean absolute reflectivity of about 33% is measured, in contrast to the roughly 90% reflectivity of bulk silver. Using the expression for the normal reflection at an interface *R* = ∣(1 − *n*_eff_)/(1 + *n*_eff_)∣^2^, we can estimate the effective refractive index of the printed silver as *n*_eff_ = 3.697 + 0.126*i* at a wavelength of 532 nm.

Furthermore, we can identify the volume fraction of silver in the fabricated devices by applying the Maxwell-Garnett effective medium model^30^. This model describes the effective permittivity of a dielectric material with metallic inclusions using the formula *ε*_eff_ = *ε*_g_[*ε*_g_ + 1 + 2*f*/3(*ε*_s_ − *ε*_g_)]/[*ε*_g_ + 1 − *f*/3(*ε*_s_ − *ε*_g_)]. Here, *ε*_g_ and *ε*_s_ are the bulk permittivities of the gel and silver, respectively, and *f* denotes the volume fraction of the silver particles. We find a volume fraction of *f* = 0.39, which suggests that a slightly higher volume fraction (up to ~ 0.74), and therefore reflectivity, is possible.

Additionally, Fig. 4b shows the measured relative reflectivity of square patches printed with different laser energies (60 nJ to 190 nJ), corresponding to laser power (75−120 mW) and scanning speeds (15−30 mm/s). The optical reflection is clearly affected by the printing energy, which allows us to control the relative reflection down to ~40%.

For low densities, losses become low enough to measure an appreciable transmission. Figure 4b shows the transmission for a low-density silver patch. A clear Fabry-Pérot interference pattern emerges from the silver thin-film. We estimate the effective index of the low-density silver for a narrowband spectral width $T={(1-R)}^{2}/[{(1-R)}^{2}+4R{\sin }^{2}(\delta /2)]\exp (-\alpha {L}_{\mathrm{fi}lm})$, where $R=[{({n}_{{\rm{gel}}}-{n}_{\mathrm{fi}lm})}^{2}+{k}_{\mathrm{fi}lm}^{2}]/[{({n}_{{\rm{gel}}}+{n}_{\mathrm{fi}lm})}^{2}+{k}_{\mathrm{fi}lm}^{2}]$ is the Fresnel reflection, *δ*/2 = *ω*/*c*_0_*n*_film_*L*_film_ is the accumulated phase shift, *α* = 4*πk*_film_/λ is the absorption coefficient, λ is the wavelength, *L*_film_ the silver patch thickness, *c*_0_ the speed of light in vacuum, *n*_gel_ = 1.35 the gel index, and *n*_eff_ = *n*_film_ + *ik*_film_ the thin film complex index of refraction. Fitting for the index and thickness, we find *n*_eff_ = 2.14 + *i*0.0009 for a film thickness *L*_film_ = 21 *μ*m. Having characterized the basic optical properties of nanomaterials fabricated with ImpFab, we now turn our attention to the realization of nanophotonic devices.

### 2D and 3D photonic crystals

**Fig. 5:**
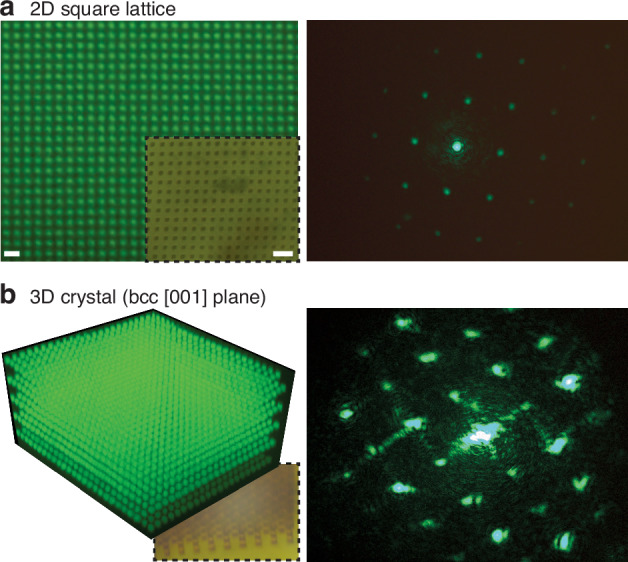
Photonic crystals with corresponding diffraction patterns. **a** 2D square lattice with periodicity *a* = 700 nm and cube width 0.5 *a*. Fluorescence image taken after conjugating the printed area with biotin-NHS particles and partial shrinking with MgCl_2_ (3-4x shrink ratio). The inset shows a bright field image of a crystal with *a* = 2.4 *μ*m and cube width 0.4 *a*. The right panel shows the corresponding diffraction pattern. Scale bar for fluorescence image is 2 *μ*m, and 5 *μ*m for the bright field image. **b** 3D body-centered cubic (bcc) crystal with *a* = 2.4 *μ*m

**Fig. 6:**
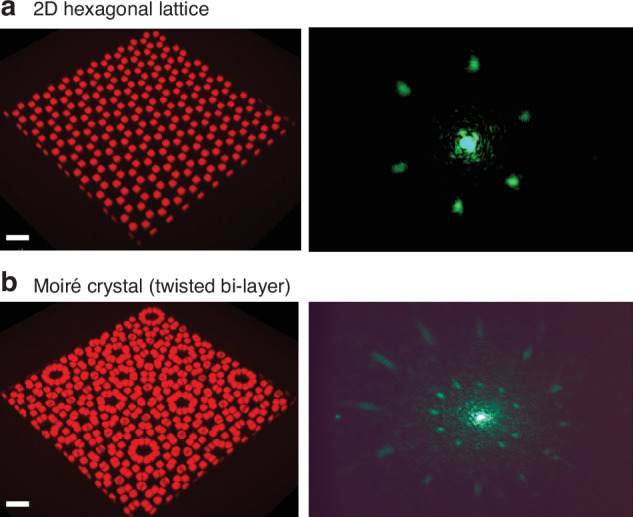
Photonic crystals with corresponding diffraction patterns. **a** 2D hexagonal lattice crystal with primitive translation vector *a* = 4.5 *μ*m. The right panel shows the corresponding diffraction pattern. **b** Twisted bi-layer hexagonal lattice forming a moiré crystal. Diffraction pattern with characteristic 12-fold symmetry of quasicrystal (right panel). Scale bars are 20 *μ*m

In this section, we show how ImpFab can realize 2D and 3D photonic crystals formed by silver meta-atoms with various crystal lattices. Figures 5 and 6 show the fluorescence and optical microscope images of fabricated photonic crystals, alongside their corresponding diffraction patterns. The diffraction measurements were performed under narrow-band illumination at 520 nm, derived from a spectrally filtered supercontinuum laser.

Figure 5a left panel shows the fluorescence image of a fabricated 2D photonic crystal with square lattice, with periodicity *a* = 700 nm and width 0.5 *a*. The inset of Fig. 5a shows a bright field microscope image of a crystal structure consisting of silver cubes arranged in a lattice with periodicity *a* = 2.4 *μ*m and width 0.4 *a*. The corresponding diffraction pattern is shown in the right panel of Fig. 5a, demonstrating a clear fourfold symmetry. This symmetry reflects the equidistant arrangement of meta-atoms along two perpendicular axes in the lattice. Diffraction peaks manifest at locations corresponding to the reciprocal lattice vectors, affirming the characteristic periodicity and integrity of the square lattice structure. The three-dimensional body-centered cubic (bcc) crystal shown in Fig. 5b yields a similar diffraction pattern, stemming from its three-dimensional nature and the specific meta-atom arrangement of the bcc structure. The right panel of Fig. 5b shows the diffraction pattern projected along the [001] plane.

Figure 6a shows a single-layer hexagonal lattice, displaying a sixfold symmetry in its diffraction pattern. This pattern offers a direct visualization of the inherent hexagonal arrangement of the meta-atoms within the lattice structure. The ImpFab method’s versatility and precision make it possible to create more complex structures such as twisted bilayer hexagonal “moiré” crystals. This is achieved by printing a stack of two separate hexagonal lattices with a twist angle. The interference of the two twisted layers effectively introduces a new length scale into the system, the moiré wavelength, giving rise to a larger *superlattice* moiré structure, seen in Fig. 6b. The moiré wavelength^31^ can be expressed as *Λ*_*m*_ = *a*/[2*sin*(*θ*/2)], where *a* is the lattice period and *θ*_*m*_ is the relative rotation between layers. The interaction between layers provides opportunities for complex band engineering, such as photonic flat bands^32^, as well as strong light localization and chirality^10,33^.

The realized structure, shown in Fig. 6b, has a twist angle *θ*_*m*_ = 30^∘^, *a* = 20 *μ*m and *z* spacing of ~1 *μ*m, resulting in a moiré wavelength of approximately *Λ*_*m*_ = 8.7 *μ*m. For this angle, an intriguing phenomenon is observed: the diffraction pattern displays a twelve-fold symmetry, analogous to quasicrystals, materials known for their unique diffraction patterns and lack of periodicity^34,35^. This is in stark contrast to the single layer shown in 6a. The additional Bragg peaks in the diffraction pattern originate from the second twisted layer and are positioned according to the inverse of the moiré wavelength, yielding the observed twelve-fold symmetry. The emergence of this quasicrystalline-like diffraction pattern from a bilayer system with crystalline layers demonstrates the fascinating possibilities presented by the control and manipulation of layered nanophotonic structures^10^.

### Quasicrystals

Quasicrystals lack periodicity but do exhibit long-range orientational order. This unique property of quasicrystals, marked by non-repetitive patterns that still maintain a certain degree of order, poses a challenge for most conventional 3D fabrication techniques. However, ImpFab, with its nanoscale precision and flexibility of 3D structure and material density, is well-equipped to handle such complexity.

**Fig. 7:**
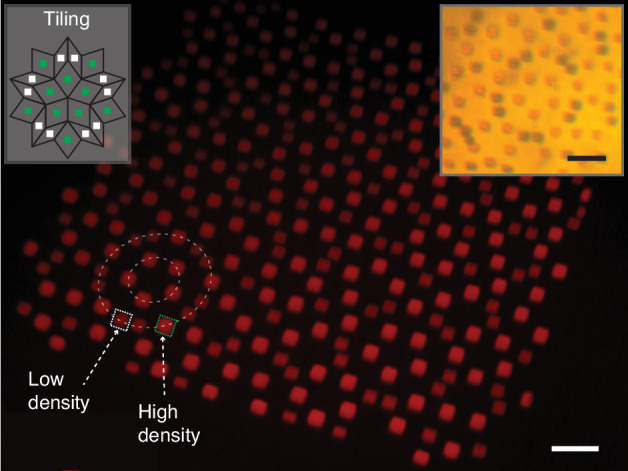
Optical structures with modulated unit cell fabricated with ImpFab 2D Penrose quasicrystal with unit cells having two distinct material densities (e.g., gain/loss). White scale bar is 20 *μ*m, black scale bar is 6 *μ*m

Figure 7 shows an optical Penrose quasicrystal, a canonical example of a 2D quasicrystal, consisting of an aperiodic two-tiling structure exhibiting fivefold rotational symmetry. Silver meta-atoms of distinct densities were placed at the center of each of the two tiles, as illustrated in the inset of Fig. 7. This serves as an example of an optical structure assembled from constituents with precisely controlled loss. In future experiments, using an optically patterned fluorescent dye, one could pump the dye to obtain gain within the same structure. By printing two sets of dyes, one set can be used as gain, while the other, with an overlapping spectral profile can be used as loss, effectively creating a non-Hermitian optical structure.

**Fig. 8:**
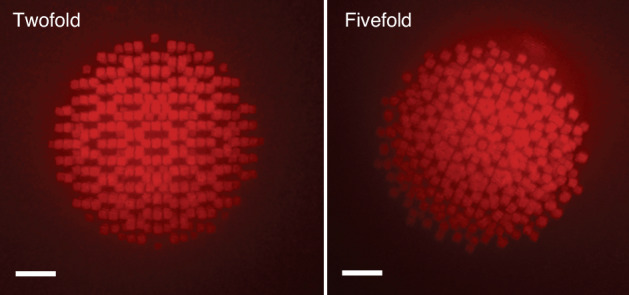
3D quasicrystals fabricated with ImpFab. Reconstructed 3D icosahedral quasicrystal. The left and right panel shows fluorescence projection images for twofold- and fivefold-symmetric axes, respectively. Scale bars are 50 *μ*m

Figure 8 shows 2D projections of a fabricated 3D icosahedral quasicrystal along different symmetry axes (two- and five-fold), with characteristic spacing *a* = 1.4 *μ*m and width 0.5 *a*. Such noncrystallographic symmetries naturally arise from the interpretation of quasicrystals as projections of higher-dimensional cubic lattices onto irrationally oriented hyperplanes, known as the cut-and-project method^36,37^. The Penrose and icosahedral quasicrystals are projections from 5D and 6D cubic lattices onto 2D and 3D, respectively, with orientations related to the golden ratio, which introduces the fivefold rotational symmetry. Constraints in our printing system resulted in a periodic *z*-layer stack, necessitating artificial compression of the 3D fluorescence image along *z* to discern the symmetry patterns more efficiently.

## Discussion

In this work, we employed the versatility and spatial controllability of ImpFab to nanofabricate photonic crystals and quasicrystals in 2D and 3D. These results pave the way for the exploration of a new realm of multi-dimensional photonic structures with non-trivial symmetries and their associated properties, offering vast potential for innovative applications.

Unlike periodic structures, which are marked by their long-range translational symmetry, quasicrystals are highly isotropic, owing to their non-crystalline symmetries. Consequently, they have been shown to host complete photonic band gaps at lower dielectric contrasts, potentially enabling more efficient control and manipulation of light with comparatively relaxed material constraints^38^. Moreover, photonic quasicrystals often exhibit interesting localization physics, such as strongly localized states, localization/delocalization transitions and mobility edges^39–41^, which hold practical implications for manipulating light flow in complex photonic systems. This includes the formation of high *Q*/*V* nanocavities^42^ and enhancing light transport in the presence of disorder^43^. Additionally, the unique properties of photonic quasicrystals can be harnessed to mitigate crosstalk in fiber arrays, a major challenge in both optical communications and endoscopy^44^, and to open up new avenues for lasing applications^45^. A method to realize three-dimensional photonic crystals and quasicrystals and twisted multilayer systems with arbitrary material properties would enable the exploration of new frontiers in photonics inspired by condensed matter physics^34,35,46,47^.

Gain and loss are ubiquitous in photonics, with the former being essential for light generation and amplification, and the latter typically viewed as a detrimental property. However, recent explorations in non-Hermitian photonics have revealed the potential to harness unique properties by judiciously distributing gain and loss within a photonic system^17,48,49^. Non-Hermitian photonic systems exhibit exceptional points and novel topological effects^50^ which have been proposed for a variety of applications, including quantum information processing^51^, sensing^52^, lasing^22,53^, and robust light transport^54,55^. ImpFab may enable the fabrication of a new class of non-Hermitian devices based on photonic crystals, which are anticipated to exhibit several unique properties, including all-angle supercollimation, the PT-superprism effect, and threshold-less PT transitions^56,57^. As such, this fabrication technique has the potential to significantly expand the capabilities of photonic devices. We note that the experimental realization and performance testing of photonic structures incorporating gain and loss using fluorescent dyes, such as PT-symmetric systems, represent an important future direction for extending the capabilities of Implosion Fabrication.

## Materials and methods

### Two-Photon photopatterning

The photopatterning of all structures was carried out using a custom-built two-photon lithography system based on a Mai Tai Ti:sapphire femtosecond laser (Spectra-Physics). The laser operated at a center wavelength of 780 nm, with a pulse width of 100 fs and a repetition rate of 80 MHz. The beam was expanded to fill the back aperture of a 20 × water-immersion objective (numerical aperture = 1.00, working distance = 2.80 mm), ensuring optimal focusing and resolution. The optical power delivered to the sample ranged from 75 mW to 120 mW, adjusted according to the desired feature size and nanoparticle deposition density. The system provided a 600 *μ*m field of view at 2048 pixels, corresponding to a pixel size of 293 nm. A dwell time of 0.8 *μ*s per pixel yielded an effective scan speed of approximately 370 mm/s. Photopatterning parameters, including average laser power, dwell time, and z-step size, were optimized to achieve high structural fidelity.

The three-dimensional structures were defined digitally as a stack of two-dimensional binary masks, each corresponding to a planar cross-section of the desired geometry. These masks were discretized and translated into position and amplitude modulation commands for the laser scanning system during two-photon lithography (TPL). The femtosecond laser is tightly focused into the hydrogel at specific depths and scanned through the hydrogel to activate photochemical reactions. The patterning voxel, the smallest addressable volume pixel, is determined by the numerical aperture (NA) of the objective, laser power, and scanning speed. These parameters were experimentally calibrated to ensure high patterning fidelity and consistent material deposition across layers.

### Fluorescence Imaging

All fluorescence imaging was performed using a Perkin Elmer spinning disk confocal microscope (CSU-10 Yokogawa) equipped with a Hamamatsu Orca-ER cooled CCD camera. Excitation was provided by continuous-wave (CW) laser lines chosen according to the fluorophore under study. Imaging was carried out using a Nikon N40XLWD-NIR 40 × water-immersion objective (NA = 1.15, working distance = 0.59–0.61 mm). The illumination power was adjusted to maximize fluorescence signal while avoiding photobleaching, and polarization control was not required for these measurements. For Cy3, the excitation wavelength was 561 nm, and fluorescence emission was collected in the range of 570-650 nm using appropriate bandpass filters.

## Supplementary information

Supplementary Information

## Data Availability

The data and codes that support the plots within this paper and other findings of this study are available from the corresponding authors upon reasonable request. Correspondence and requests for materials should be addressed to Y. S. (yannick.salamin@ucf.edu).
