# Supplementary material for: Three-dimensional nanophotonics with spatially modulated optical properties
Source: Light Sci Appl. 2026 Mar 3;15:145. doi: 10.1038/s41377-025-02166-5 (PMC12957445; doi:10.1038/s41377-025-02166-5)
Supplement: Supplementary file 1 — Supplementary Information [file 41377_2025_2166_MOESM1_ESM.pdf]

Supplementary Information for

Three-Dimensional Nanophotonics with Spatially Modulated Optical Properties

Yannick Salamin[1,2,3*†], Gaojie Yang[4†], Brian Mills[1], André Grossi Fonseca[2], Charles Roques-Carmes[1,5], Quansan Yang[4,6], Justin Beroz[6], Steven E. Kooi[7], Marc de Miguel Comella[1,8], Kiran Mak[1], Sachin Vaidya[1,2], Daniel Oran[9], Corban Swain[4,10], Yi Sun[1], Shai Maayani[1], Jamison Sloan[1], Amel Amin Elfadil Elawad[1], Josue J. Lopez[1], Edward S. Boyden[4,10,11,12,13,14], and Marin Soljačić[1,2]

[1]Research Laboratory of Electronics, MIT, Cambridge, 02139, MA, USA.

[2]Department of Physics, MIT, Cambridge, 02139, MA, USA.

[3]CREOL, The College of Optics and Photonics, University of Central Florida, Orlando, 32816, FL, USA.

[4]McGovern Institute for Brain Research, MIT, Cambridge, 02139, MA, USA.

[5]Ginzton Laboratories, Stanford University, Stanford, 94305, CA, USA.

[6]Department of Mechanical Engineering, MIT, Cambridge, 02139, MA, USA.

[7]Institute for Soldier Nanotechnologies, 500 Technology Square, Cambridge, 02139, MA, USA.

[8]CFIS, Universitat Politècnica de Catalunya, Barcelona, 08028, Spain.

[9]Irradiant Technologies, Cambridge, 02139, MA, USA.

[10]Department of Biological Engineering, MIT, Cambridge, 02139, MA, USA.

[11]Department of Brain and Cognitive Sciences, MIT, Cambridge, 02139, MA, USA.

[12]Howard Hughes Medical Institute, MIT, Cambridge, 02139, MA, USA.

[13]K. Lisa Yang Center for Bionics, MIT, Cambridge, 02139, MA, USA.

[14]Center for Neurobiological Engineering, MIT, Cambridge, 02139, MA, USA.

*Corresponding author(s). E-mail(s): yannick.salamin@ucf.edu

[†]These authors contributed equally to this work.

Supplementary Note 1: Controllability of Structural Parameters and Shrinkage Isotropy

To investigate the controllability of structural parameters and their corresponding optical responses in Implosion Fabrication (ImpFab), we performed additional experiments using silver diffraction gratings as representative photonic structures with well-defined and tunable optical signatures. Metallic gratings with designed periods ranging from approximately 570 nm to 1700 nm were fabricated (Figure S1a), enabling systematic variation of the photonic structure's periodicity. The inset of Figure S1a shows an optical microscopy image of a silver grating with a groove size of about 850 nm, demonstrating that the patterned geometry is preserved through both silver deposition and hydrogel shrinkage.

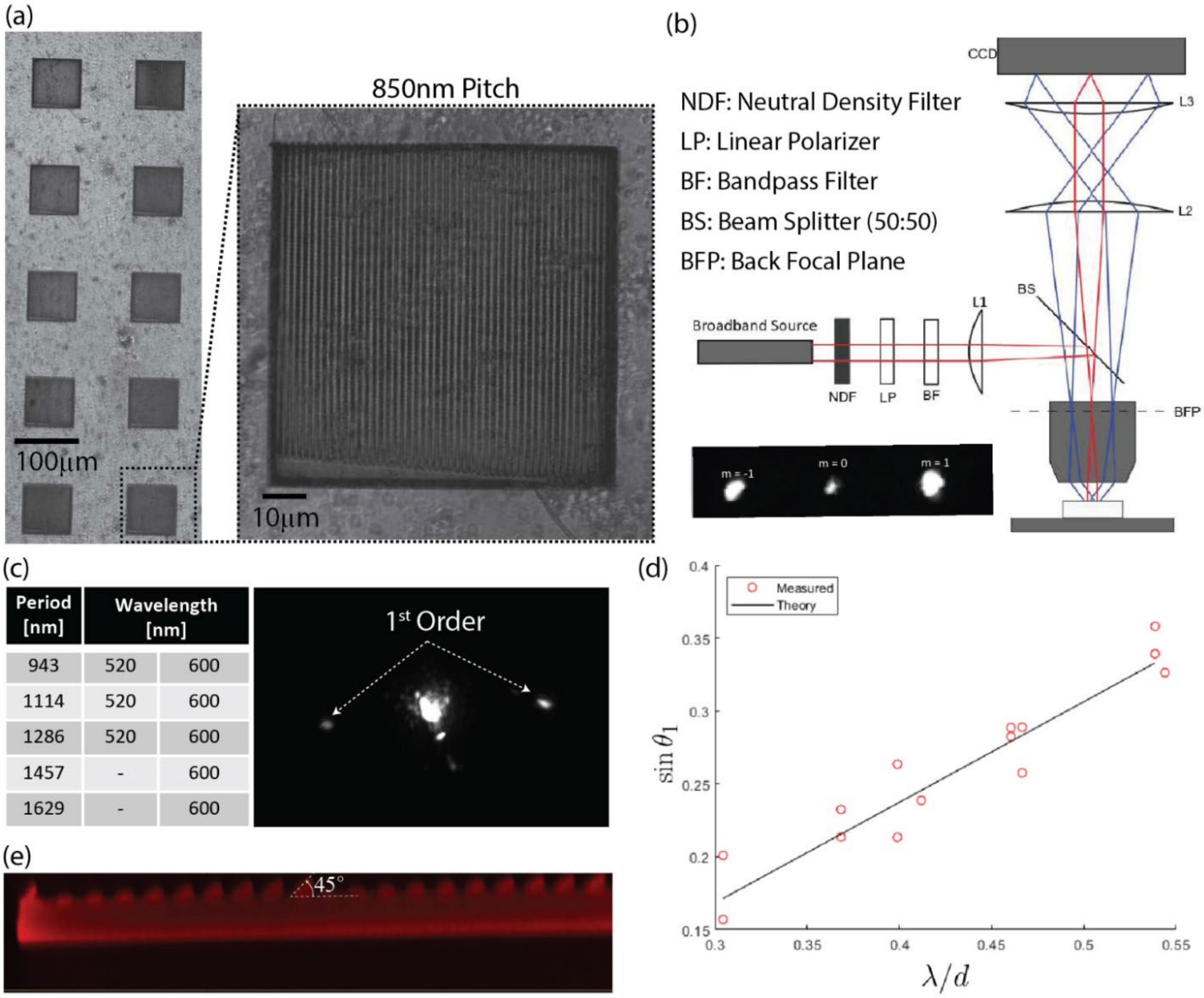

Figure S1: (a) Optical microscopy image of silver gratings with varying pitch (approx. 570 nm to approx. 1700 nm). (b) Fourier microscopy setup; a lens focuses an initial beam onto the back focal plane of an objective, spatially resolving diffraction spots from the grating, which is then projected onto a CCD. (c) An example of first-order optical diffraction was observed with a narrow band light source. The distance between the diffraction spots recorded with the CCD camera allows calculating the diffraction angle. (d). Plot of measured diffraction angle as a function of measurement wavelength λ and grating pitch d. The black line shows the expected slope governed by the grating equation. (e) Fluorescence image of a sawtooth-like structure with varying heights after uniform isotropic shrinkage (~5×). The preserved geometry and consistent feature scaling across the pattern demonstrate the uniformity of the shrinkage process.

Optical characterization was carried out using a custom-built Fourier microscopy system (Figure S1b) calibrated with commercial gratings of known periodicity. First-order diffraction angles were measured under narrow-band illumination at 520 nm and 600 nm. An example image of the diffraction pattern for narrow-band illumination is shown in Figure S1c. The measured diffraction angles closely match theoretical predictions from the grating equation (Figure S1d), further confirming the isotropic nature of the shrinkage process.

We further examined shrinkage isotropy by fabricating additional periodic structures, including sawtooth-like gratings with varying heights, and imaging them after shrinkage using fluorescence microscopy (Figure S1e). The preserved geometry and uniform feature scaling across these patterns confirm that the shrinkage process is isotropic. While slight variations in shrinkage rate may occur due to local differences in material density or functionalization, our results indicate that such effects are minimal and have negligible impact on the optical performance of the fabricated structures.

Supplementary Note 2: Optimization of Redox Reaction Process and Nanoparticle Deposition Density

For the fabrication of silver-based photonic structures, achieving a high density of metallic nanoparticles is essential for optimal optical performance. To this end, we optimized the patterning chemistry to increase the density of patterned molecules available for nanoparticle conjugation and deposition. Sulfo-Cyanine3 (Cy3) was used in the main manuscript for two-photon lithography (TPL) due to its shorter absorption wavelength. However, for process optimization, we investigated chromophores with higher two-photon absorption cross-sections, including Sulfo-Cyanine5 (Cy5) and fluorescein (FGA).

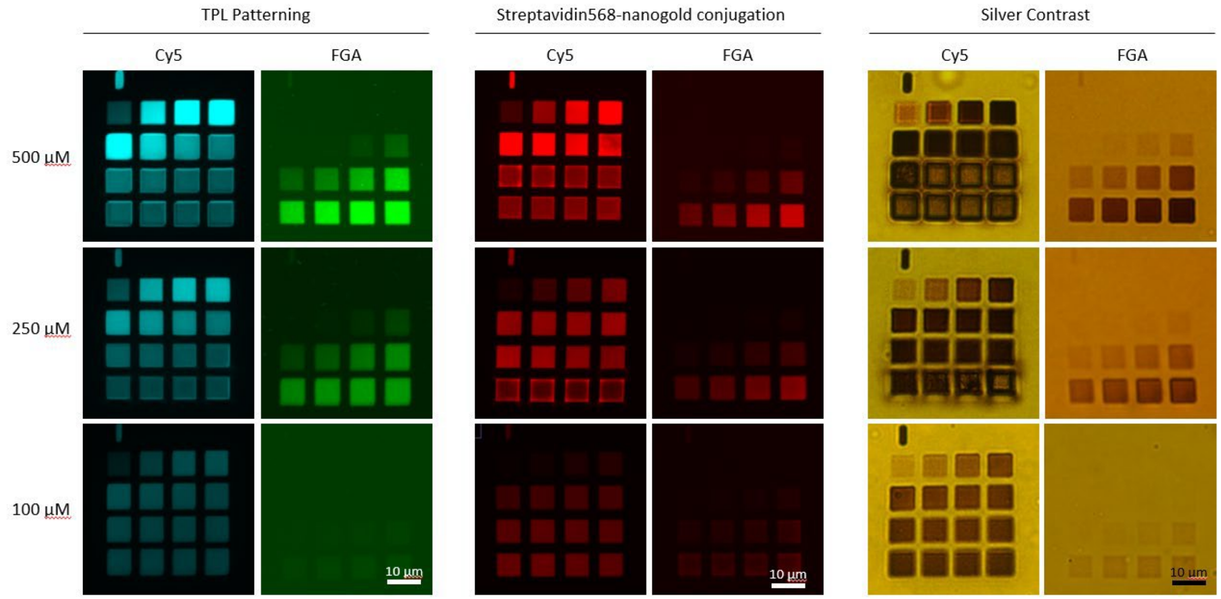

Figure S2: Performance comparison of Cy5 and FGA across different concentrations for TPL patterning, streptavidin568-nanogold conjugation, and silver intensification. Gradient patterns were generated under varying laser powers, with increasing laser intensity.

Figures S2 and S3 present a comparative study of Cy5 and FGA performance in TPL patterning, nanoparticle conjugation, and silver intensification. Fluorescence and wide-field imaging revealed that Cy5

exhibited superior patterning performance compared to FGA. The laser intensity required to achieve maximum activation for Cy5 was approximately 18 mW, which is about five times lower than the ~90 mW required for FGA. This lower activation threshold resulted in a higher molecular density in the patterned regions, which in turn increased nanoparticle binding efficiency. Subsequent conjugation with streptavidin568-nanogold and intensified silver deposition confirmed that patterns written with Cy5 yielded a greater density of metallic nanoparticles, as shown in Figures S2 and S3. Quantitative measurements demonstrated higher fluorescence intensity in Cy5 patterns compared to FGA at equivalent laser powers, greater silver contrast in the patterned regions, and enhanced optical absorption due to the increased particle coverage.

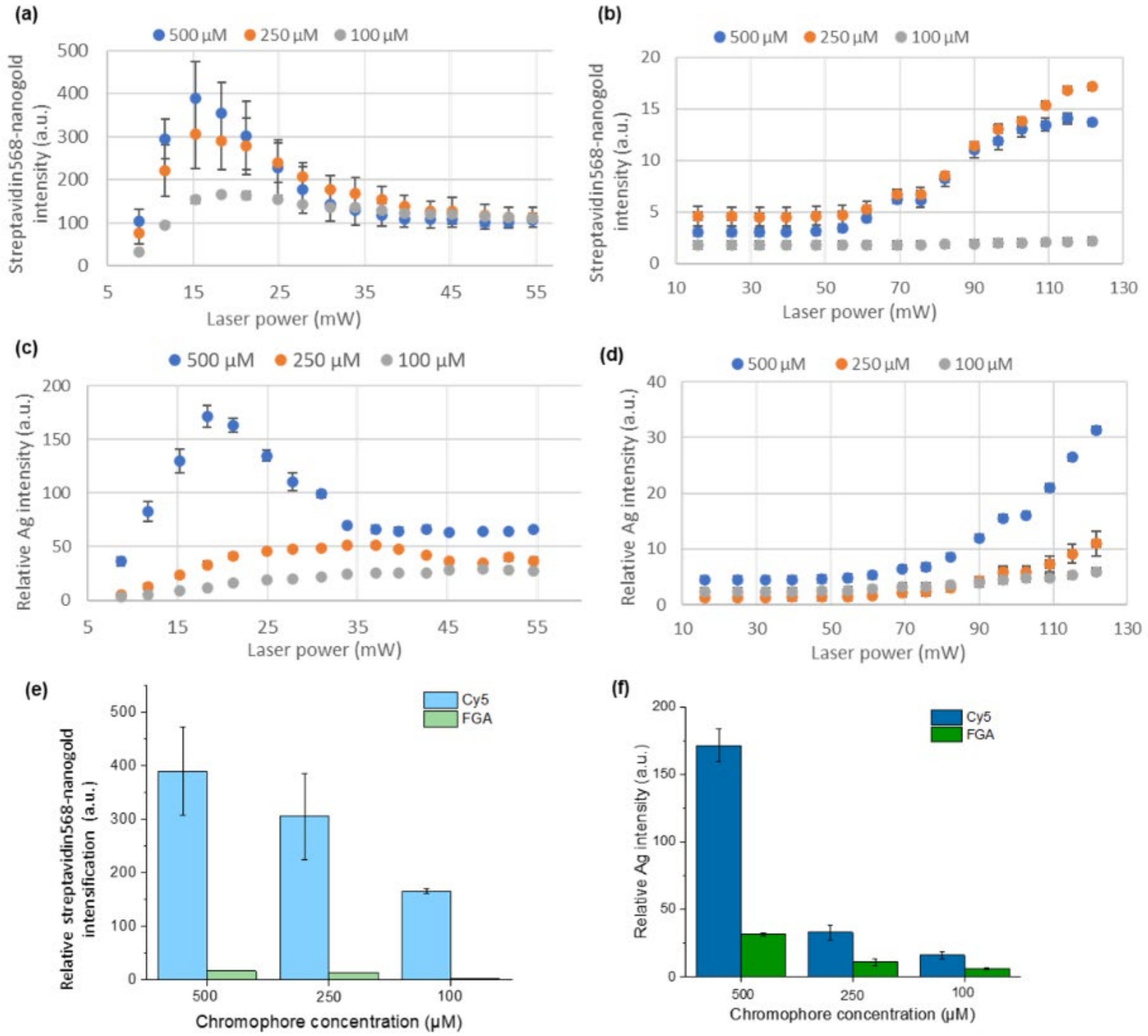

Figure S3: The quantitative analysis of Cy5 and FGA performance of patterning, conjugation, and nanoparticle deposition. (a) and (b) Fluorescence intensity of streptavidin568-nanogold square patches written at different laser power resulting in different nano-gold particle concentrations (gradient) for Cy5 and FGA, respectively. (c) and (d) Silver contrast with different concentrations by different laser power for Cy5 and FGA, respectively. (e) and (f) Comparison of streptavidin568-nanogold conjugation and contrasted silver absorption between Cy5 and FGA structures with the highest intensity.

To further improve material deposition density, a hydrophobic functionalization strategy was implemented. Hydrophobic lipids were conjugated directly to the patterned chromophores, creating regions of controlled hydrophobicity that maintained the original geometry while selectively attracting hydrophobic materials. This method enabled more efficient infiltration and deposition of nanoparticles into the patterned areas. Compared to traditional site-by-site conjugation methods, hydrophobic functionalization significantly increased the number of nanoparticles deposited, as evidenced by a measurable increase in optical absorption (Figure S4a). SEM imaging confirmed that the gold nanoparticles in these regions were densely packed and interconnected (Figure S4b), indicating a high activation and functionalization rate of the chromophores and, consequently, greater material deposition.

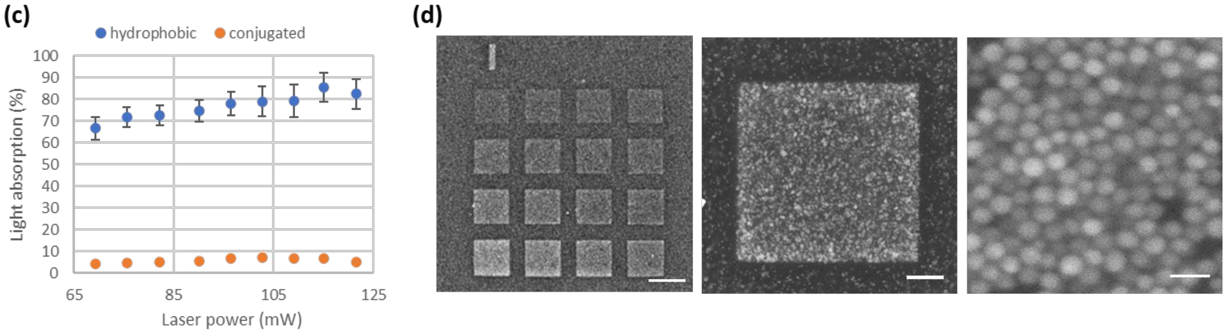

Figure S4: (a) Light absorption mediated by gold nanoparticle deposition as a function of laser power, for hydrophobic lipid–modified chromophores (blue) and conventional conjugation (orange). (b) SEM images showing patterned regions with dense, uniform gold nanoparticle. (Right) High-magnification SEM image reveals interconnected nanoparticles within the patterned area, confirming high chromophore activation and efficient material deposition. Scale bar: left = 10 µm, middle = 1 µm, right = 50 nm.

Supplementary Note 3: Two-Photon Photopatterning and Fluorescence Imaging

The photopatterning of all structures was carried out using a custom-built two-photon lithography system based on a Mai Tai Ti:sapphire femtosecond laser (Spectra-Physics). The laser operated at a center wavelength of 780 nm, with a pulse width of 100 fs and a repetition rate of 80 MHz. The beam was expanded to fill the back aperture of a 20× water-immersion objective (numerical aperture = 1.00, working distance = 2.80 mm), ensuring optimal focusing and resolution. The optical power delivered to the sample ranged from 75 mW to 120 mW, adjusted according to the desired feature size and nanoparticle deposition density. The system provided a 600 µm field of view at 2048 pixels, corresponding to a pixel size of 293 nm. A dwell time of 0.8 µs per pixel yielded an effective scan speed of approximately 370 mm/s. Photopatterning parameters, including average laser power, dwell time, and z-step size, were optimized to achieve high structural fidelity.

The three-dimensional structures were defined digitally as a stack of two-dimensional binary masks, each corresponding to a planar cross-section of the desired geometry. These masks were discretized and translated into position and amplitude modulation commands for the laser scanning system during two-photon lithography (TPL). The femtosecond laser is tightly focused into the hydrogel at specific depths and scanned through the hydrogel to activate photochemical reactions. The patterning voxel, the smallest addressable volume pixel, is determined by the numerical aperture (NA) of the objective, laser power, and scanning speed. These parameters were experimentally calibrated to ensure high patterning fidelity and consistent material deposition across layers.

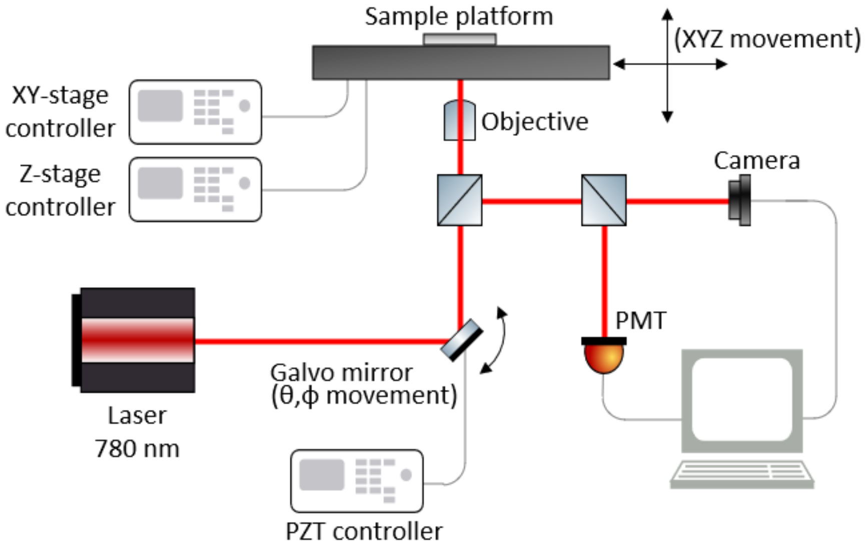

Figure S5: Simplified photopatterning setup.

All fluorescence imaging was performed using a Perkin Elmer spinning disk confocal microscope (CSU-10 Yokogawa) equipped with a Hamamatsu Orca-ER cooled CCD camera. Excitation was provided by continuous-wave (CW) laser lines chosen according to the fluorophore under study. Imaging was carried out using a Nikon N40XLWD-NIR 40× water-immersion objective (NA = 1.15, working distance = 0.59–0.61 mm). The illumination power was adjusted to maximize fluorescence signal while avoiding photobleaching, and polarization control was not required for these measurements. For Cy3, the excitation wavelength was 561 nm, and fluorescence emission was collected in the range of 570–650 nm using appropriate bandpass filters.
